# The discovery of the use of magnetic navigational information

**DOI:** 10.1007/s00359-021-01507-0

**Published:** 2021-09-02

**Authors:** Roswitha Wiltschko, Wolfgang Wiltschko

**Affiliations:** Fachbereich Biowissenschaften, Goethe-Universität Frankfurt, Max-von-Laue-Straße 13, 60438 Frankfurt am Main, Germany

**Keywords:** Geomagnetic field, Magnetic compass, Magnetic conditioning, Magnetic map, Sign posts

## Abstract

The magnetic field of the Earth provides animals with various kinds of information. Its use as a compass was discovered in the mid-1960s in birds, when it was first met with considerable skepticism, because it initially proved difficult to obtain evidence for magnetic sensitivity by conditioning experiments. Meanwhile, a magnetic compass was found to be widespread. It has now been demonstrated in members of all vertebrate classes, in mollusks and several arthropod species, in crustaceans as well as in insects. The use of the geomagnetic field as a ‘map’ for determining position, although already considered in the nineteenth century, was demonstrated by magnetically simulating displacements only after 2000, namely when animals, tested in the magnetic field of a distant site, responded as if they were physically displaced to that site and compensated for the displacement. Another use of the magnetic field is that as a ‘sign post’ or trigger: specific magnetic conditions elicit spontaneous responses that are helpful when animals reach the regions where these magnetic characteristics occur. Altogether, the geomagnetic field is a widely used valuable source of navigational information for mobile animals.

The earth is surrounded by a magnetic field that can provide useful information for orientation and navigation. Its vector quality is the basis for a magnetic compass, and its intensity and inclination can serve as components for determining positions. We humans make use of the geomagnetic field for direction finding already since hundreds of years, using a technical instrument, a compass. Yet it took quite a while until it was discovered that animals, too, can use magnetic information for orientation and navigation, although this possibility was already discussed in the nineteenth century (von Middendorff 1859; Viguier 1882). Today, we know that many animals—in particular vertebrates and arthropods—respond to various magnetic stimuli, even if it is not always clear what property of the magnetic field is important and for what the animals use it in nature. Here, we will briefly summarize how the role of the geomagnetic field in animal navigation was discovered, what the initial hurdles were and what we know about its use.

## The avian magnetic compass

First experimental data showing that animals can use the magnetic field for directional orientation were published by the Frankfurt group in the mid-1960s (W. Wiltschko and Merkel 1966): a nocturnally migrating songbird species, the European Robins, *Erithacus rubecula* (Turdidae), oriented its migratory activity in a test cage in its normal migratory direction, and when magnetic North was deflected by Helmholtz coils, it shifted its headings accordingly (see also W. Wiltschko 1968). This was the first demonstration that animals could use the geomagnetic field for a compass.

### Initial problems with demonstrating a magnetic compass of birds

These early findings met with great skepticism. For many researchers, it was hard to believe that birds would have a sensory capacity alien to us humans. Also, there was the feeling that after the discovery of the sun compass (Kramer 1952) and the nocturnal orientation by stars (Sauer 1957) there was no room for another compass. More important, however, was that first attempts to find evidence for a magnetic sensitivity in birds had already produced negative results. Prominent scientists like Erwin Stresemann in Germany and Donald Griffin in the USA had considered the geomagnetic field as potential useful orienting cue, but failing to find evidence for magnetoreception, they decided that a sensitivity of birds to magnetic cues could be largely excluded (Stresemann 1935; Griffin 1952, 1955). Even after the first positive results on migratory birds had been published (W. Wiltschko and Merkel 1966; W. Wiltschko 1968), the skepticism remained, because it proved hard to reproduce these findings. There were a number of reports showing that night-migrating birds had shown oriented behavior in migratory direction in test cages (e.g. Sauer 1957; Mewaldt et al. 1964: Emlen 1967 a.o.), but in these cases, celestial cues—the natural stars or a naturally-looking planetarium sky—had been visible. It was generally accepted that might-migrating birds could orient by stellar cues, but the claim of the Frankfurt group that night-migrating birds could also orient ‘non-visually’, i.e. without stars (Fromme 1961), could not be confirmed by other authors (e.g. Perdeck 1963; Wallraff 1966).

There were several reasons for these contradictory findings; they included handling of birds, the nature of the test fields, statistical treatment of the data and the construction of the test cages. In the early 1960s, a standard method of treating circular data had not yet been established. The distribution of the bird’s activity in a test cage often differed only slightly from a uniform distribution, with the preference of the most active sector of the cage being mostly very small. When statistical tests were applied to the distribution of this activity, a significant deviation from random was usually not found, and it was discarded as “not oriented”. Such statistical testing, however, cannot be applied, because the moves of the test birds in the cage are not independent from each other. It should also be considered that the distribution of the bird’s activity is not only controlled by orientation, but also partly reflects the bird’s response of being in captivity and its urge to express its migratory activity in a limited enclosure where flying is impossible. When the activity of each test was summarized into a single heading, these headings were found to concentrate in migratory direction, and their distribution could be tested by the Rayleigh Test for significance (Batschelet 1965). Later, a second order statistic was mostly applied, based on the mean headings of the individual birds calculated from several tests.

Another initial problem was the design of the test cages. They all were circular (or octagonal), but their proportions varied, and so did the arrangement of perches inside—it ranged from a circular perch to various numbers of tangentially or radially arranged perches. The positive results of the Frankfurt group had been obtained in an octagonal cage with eight radially positioned double perches; the cage was about 40 cm high and allowed the test bird to move rather freely inside. Wallraff (1966) had tried various cage arrangements and had published negative results; he borrowed this cage and, testing robins, he found that the birds were oriented in their natural migratory direction in spring as well as in autumn. The directional preference in each test was rather weak, with only little difference in the activity on the eight perches. Yet the slightly more activity on some perches resulted in nightly headings that clustered in the migratory direction, and the mean vector calculated from them was statistically significant (Wallraff 1972), thus confirming migratory orientation without celestial cues.

Emlen, who had observed oriented behavior of Indigo Buntings, *Passerina cyanea* (Emberizidae*)*, under a planetarium sky in a funnel-shaped cage (Emlen 1967), had failed to find orientation without stars (Emlen 1970). In joint spring experiments, using the Frankfurt cages and funnel-shaped cages, the combined data showed that in natural geomagnetic field, the birds showed a weak, but persistent preference of their northern migratory direction; in a field of equal intensity, but with North shifted by 120° to ESE, they shifted their headings accordingly (Emlen et al. 1976). These findings were the first to confirm that migratory birds could orient using directional information from the magnetic field.

### A stimulus with different characteristics

The skepticism about the sensitivity of birds for the magnetic field remained, however, because it seemed more or less impossible to condition animals to magnetic stimuli. Various techniques—cardiac and operant conditioning, with rewards or shock avoidance—and different types of magnetic stimuli—changes in direction, changes in intensity, or both, low frequency fields, etc.—had been used, mostly without success (see Griffin 1982). With pigeons alone, two positive results—a cardiac conditioning by Reille (1968) and an operant study by Bookmann (1977)—are up against 11 negative ones in various studies (for a list, see R. Wiltschko and Wiltschko 1996). Guided by the conviction that “we know of no case in which proven exteroceptive sensitivity has remained impossible to demonstrate clearly with conditioning techniques” (Moore et al. 1987, p. 117), many researchers hesitated to accept magnetic sensitivity in birds.

Several reasons contributed to the problems in conditioning. The stimuli used were usually totally unnatural, often very strong, and as a result, animals would have problems to associate these stimuli with the offered reinforcers—they do not associate every stimulus with a presented reinforcer with equal ease (see Delius and Emmerton 1978). A most important problem, however, was that many researchers apparently did not consider the context in which a magnetic compass is used and that the magnetic field differs from other stimuli in its characteristics. Light, sound and smell form the representation of an animal’s environment. Any changes are alerting, as they could indicate possible prey or potential danger—animals are prepared to respond to these changes and act accordingly. The geomagnetic field, in contrast, is used for navigation only; unless animals want to reach a certain goal, they have no need to consult it. And it never changes rapidly—thus animals are not expecting any changes. Hence, when researchers used the standard conditioning techniques, placing the bird in a conditioning chamber, and, like with the other sensory qualities, changed the magnetic field, the animals mostly did not respond—they appeared not to realize the magnetic changes. This, however, does not mean that animals cannot sense the magnetic field—it means that they do not pay attention to it in the given situation (see also R. Wiltschko and Wiltschko 1996).

When animals move, the input from the environment changes; considerable parts of the brain are devoted to neutralize this effect of self-produced motion for vision, hearing etc., leaving the representation of the environment constant, but at the same time keeping the animal alert for changes. With the magnetic field, where no changes are to be expected, it is more efficient to ignore it most of the time and call upon it only when its information is required. Hence, we may assume that an animal, when it enters a new environment, may call on its magnetic compass to learn the lay of the land, i.e. where north, east etc. lies, but afterward it can rely on this information and need not call on the magnetic compass again. Considering this, conditioning experiments have to be differently designed. The magnetic situation to be tested should be set up *before* the animal is placed into the test apparatus so that it does not experience changes in the magnetic field while within. Also, because of the normal use of magnetic information in navigation, the task is to include orientation aspects, like e.g. the animal has to move in a specific magnetic direction. This way, successful magnetic conditioning experiments were possible, and a magnetic compass could be demonstrated in non-migratory avian species, like Domestic Chickens, *Gallus gallus domesticus* (Phasanidae) (Freire et al. 2005), in Zebra Finches, *Taeniopygia guttata* (Estrildidae) (Voss et al. 2007) and homing pigeons, *Columba livia domestica* (Wilzeck et al. 2010). The magnetic compass thus appears to be a general feature of birds.

### Sensing the direction of the magnetic field in birds

For a long time, the acceptance of a magnetic orientation also suffered from the fact that there was no known sensory organ that would record magnetic directions. As an analogue to our technical compass, magnetic material aligning itself with the field lines was considered, but there seemed little room in the living organism where such alignment could take place. However, an analysis of the avian magnetic compass revealed some unexpected characteristics: it is an inclination compass, not responding to the polarity of the magnetic field, but to the (axial) course of the field lines and their inclination (W. Wiltschko and Wiltschko 1972); it works only in a functional window around the local magnetic intensity, and it requires short-wavelength light from UV to green (see R. Wiltschko and Wiltschko 2014)—this pointed to another type of mechanism. Schulten and Windemuth (1986) and later, in more details, Ritz and colleagues (2000) proposed the Radical Pair Model, a spin-chemical reaction that is based on the alignment of radical pairs in the magnetic field and their effect on the singlet/triplet ratio. As site for sensing magnetic directions, the authors proposed the eyes, because of its more or less round shape, the retina covers all spatial directions, with cryptochrome, a photo-pigment with FAD (flavin adenosine dinucleotide) as chromophore, suggested as receptor molecule (see Ritz et al. 2000 for details).

Behavioral experiments supported this model: Radio frequency fields are a diagnostic test for radical pair processes (see Ritz 2001; Henbest et al. 2004); such fields with frequencies of 0.64 MHz and above let to disorientation (Ritz et al. 2004, 2009; Kavokin et al. 2014). Cryptochrome was immuno-chemically identified in the outer segments of the UV/V cones (SWS1 receptors) in the retinae of chickens, robins and several other species (Nießner et al. 2011; Bolte et al. 2021). This indicates the eyes as site of reception for magnetic directions, even if details are not yet entirely clear (see R. Wiltschko and Wiltschko 2019; Bolte et al. 2021 for discussion). The processing of magnetic directional information in the brain is associated with the visual system, but details are not yet completely understood.

## The magnetic compass and its use in the animal kingdom

Meanwhile, the use of directional information from the magnetic field was found to be widespread among moving animals, involving members of various phyla.

### Birds

So far, more than 20 avian species have been shown to use a magnetic compass. The majority of species are night-migrating birds (for a list of migrants, see, e.g. R. Wiltschko and Wiltschko 2009), because of technical reasons: Their urge to move in migratory direction during migration season provides a solid, reliable baseline for cage experiments. Birds use their magnetic compass for orientation within their home range, and the migratory species also for orientation during migration. Indeed, it is conceivable that an efficient magnetic compass was an important precondition for some species to adapt a migratory lifestyle.

Yet a most important function of the avian magnetic compass is to provide a directional reference system: it acts as reference for recording the direction of the outward journey to obtain the home direction in inexperienced young birds (R. Wiltschko and Wiltschko 1978) and also provides the reference for the innate migratory direction in first time migrants (e.g. W. Wiltschko and Gwinner 1974; Beck and Wiltschko 1988; Shumakov 1990 a.o). Also, it is a basic reference for acquiring the information to establish the experience-based navigational mechanisms, like learning the avian sun compass, as was demonstrated for homing pigeons (W. Wiltschko et al. 1983). In migratory birds, it is used to calibrate stars and sunset cues during migration (e.g. W. Wiltschko and Wiltschko 1975a, b; W. Wiltschko et al. 1998). It probably also serves as reference for the orientation of the navigational ‘map’, the mental representation of the distribution of navigational factors that allow birds to determine the course to a goal (see, e.g. Wallraff 1974; R. Wiltschko and Wiltschko 2015).

### Other vertebrates

Several species of other vertebrates, too, have been shown to orient by magnetic field. A magnetic compass has been found in all classes of vertebrates. First reports came from Phillips (1977, 1986) for newts and salamanders, from Kalmijn (1978) for stingrays, Quinn (1980); Quinn et al. (1981) and Taylor (1986) for salmon and Karlsson (1985) and Souza and colleagues (1988) for eels. In reptiles, marine turtles were the first for which a magnetic compass was demonstrated (Lohmann 1991). In mammals, after a few observations that had suggested a magnetic compass in wood mice (e.g. Mather and Baker 1981; August et al. 1989), it was found in subterranean rodents (Burda et al. 1990) and bats (Holland et al. 2006; Wang et al. 2007). Indications for a magnetic compass in humans (Baker 1980) were controversially discussed (see Baker 1987 for reviewing the conflicting findings). Meanwhile, further observations suggesting the use of magnetic directional orientation in a number of other species from the various vertebrate groups have been published. They involved a wide variety of behaviors and methods, spontaneous behaviors, including alignments in specific magnetic directions (see e.g. Begall et al. 2013 for review), or induced directions in navigational tasks and others. Yet the use of the magnetic compass and its role in navigation in other vertebrates is not yet completely analyzed.

The magnetic compass of vertebrates does not seem to be a uniform phenomenon, however, as it involves different functional modes. Amphibians and reptiles were found to use an inclination compass like birds, but that of amphibians shows a different wave-length dependency (Phillips 1986) and that of marine turtles does not require light at all (Light et al. 1993; Lohmann and Lohmann 1993). Elasmobanchs (Newton and Kajura 2020), bony fish (Quinn et al. 1981) and mammals (Marhold et al. 1997a; Wang et al. 2007), in contrast, appear to use a compass that responds to the polarity of the magnetic field, which, in turn, implies different receptive mechanisms. That of mole-rats and bats appears to be based on magnetite (Marhold et al. 1997b; Holland et al. 2008), but details on the receptive mechanisms are still unknown.

### Mollusks and arthropods

In mollusks, the nudibranch *Tritonia diomedea* (Nudibranchia) was shown to respond to the direction of the ambient magnetic field (Lohmann and Willows 1987) by choosing a different arm of a Y-shaped maze. Magnetic compass orientation was also demonstrated in a number of arthropods (for review, see Vacha 2017): They include several crustacean species, among them beach-dwelling amphipods that orient along the axis land-sea (e.g. Arendse and Barendregt 1978; Arendse and Kruyswijk 1981; Pardi et al. 1985 a.o), and the spiny lobster *Panulirus argus* (Decapoda, Reptantia) (Lohmann 1985). Among insects, magnetic compass orientation was described in beetles like *Tenebrio* (e.g. Arendse and Vrins 1975; Vacha et al. 2008), in the comb building of the honeybees (e.g. DeJong 1982), in moths (e.g. Bakers and Mather 1982) in termites (e.g. Rickli and Leuthold 1988; Jacklyn 1992), Monarch butterflies, *Danaus plexippus* (Guerra et al. 2014), and recently also in the desert ant *Cataglyphis* (Formicidae) (Fleischmann et al. 2018), to name just a few. The functional properties of the magnetic compass in arthropods have been analyzed in very few species only, and here, too, the findings do not indicate a uniform mechanism: Spiny lobsters appear to have a polarity compass (Lohmann et al. 1995), while the beetle *Tenebrio* is reported to have an inclination compass (Vacha et al. 2008). Very little is known about the reception mechanisms.

Altogether, directional orientation by the magnetic field must be considered to be a very widespread, if not a general ability of mobile species in the animal kingdom. Animals appear to use it for a great variety of navigational tasks—it is probably involved in most tasks that require directional information. Yet it is usually not the only compass mechanisms: Many animals have been shown to additionally use directional information provided by celestial cues.

## A navigational ‘map’ including magnetic components

A navigational ‘map’ that allows birds to determine their position relative to a goal is assumed to be a directionally oriented mental representation of the distribution of navigational factors. It is based on experience and must include two or more environmental gradients (for a detailed discussion on maps, see, e.g. Wallraff 1974; R. Wiltschko and Wiltschko 2015). Animals familiarize themselves with the local course of suitable gradients within their home region and can extrapolate them beyond the familiar area; within their familiar area, they can also consider local anomalies.

Because of the gradients in intensity and inclination between the magnetic poles and the magnetic equator, the geomagnetic field can provide components for such a ‘map’. Interestingly, it was one of the first factors discussed already in the nineteenth century for long-distance navigation of birds. Viguier (1882) even developed a navigational concept based entirely on the magnetic intensity and inclination. Yet early attempts to verify a role of the magnetic field in avian navigation by letting homing pigeons fly with magnets attached did not produce convincing results (e.g. Casamajor 1927). Yeagley’s (1947) navigational concept based on the vertical component of the magnetic field and the Coriolis force also met with great skepticism, as the theoretical requirements for sensitivity seemed extremely high and his first experimental results (Yeagley 1951) did not really support the model (see, e.g. Davis 1948; Gordon 1948 a.o).

The first indication that the magnetic field was indeed involved in the navigational ‘map’ for determining the course to the goal came from the observation that displaced homing pigeons departed disoriented when released in a strong magnetic anomaly where magnetic intensity varied rapidly and irregularly (Walcott 1978). Yet the best evidence supporting a magnetic ‘map’ involves experiments with captive animals.

### Magnetically simulated displacements

After 2000, a series of experiments began to test how animals would respond when they were ‘virtually displaced’ by exposing them to the magnetic field of a distant site. An early such attempt with amphibians (Fischer et al. 2001) involved changes in inclination only, and these, with1.5° to 2°, were rather unnatural, far beyond the changes newts would ever experience in nature. The first successful experiments of simulating displacements were performed with marine animals: Spiny lobsters, *Panulirus argus*, from two different sites, tested at a site in between in the natural local geomagnetic field, were found to head towards the catching locations, and they responded in a similar way when they were not physically displaced, but tested at the catching site in magnetic fields as they occur about 400 km north and south of that site: They showed significant headings that would have brought them back from the simulated sites to the test site (Boles and Lohmann 2003). In an analogue experiment, green sea turtles, *Chelonia mydas*, caught at their feeding sites and tested in magnetic fields as they occur more than 300 km north and south of that site, also showed headings that compensated this virtual displacement (Lohmann et al. 2004), thus indicating a large-scale magnetic ‘map’ (see also Luschi et al. 2007). A recent study involving virtual ‘magnetic displacements’ suggests a magnetic ‘map’ also in bonnethead sharks, *Sphyrna tiburo* (Keller et al. 2021).

This type of experiments was also performed with migratory birds. Lesser whitethroats, *Sylvia curruca.* caught in spring in southern Sweden, showed random orientation when tested in a field north of their distribution range, but showed clear northward orientation when tested in a field as found 1000 km south of the trapping site (Henshaw et al. 2010). In a study with Australian silvereyes, *Zosterops lateralis* of Tasmanian origin during autumn migration, adult birds produced similar result, whereas juveniles (whose migration is still controlled by the innate migration program, see e.g. Berthold 1988) continued in migratory direction (Deutschlander et al. 2012). While these studies used virtual displacements along the migration route, another experiment displaced birds during spring migration almost perpendicular to the migration route. At the catching site at Rybachy (55° 09′ N, 20° 52′ E), reeds warblers, *Acrocephalus scirpaceus* (Sylviidae), showed a pronounced northeastern preference towards their breeding area in the northern Baltic states and southern Finland. After physical displacement to Zwenigorod in Russia (55° 42′ N, 36° 45′ E) the birds, tested in the local geomagnetic field, compensated for the displacement, now heading northwest (Chernetsov et al. 2008). They responded in a similar way, also showing northwesterly headings, when tested at Rybachy in the geomagnetic field of Zwenigorod (Kishkinev et al 2015). This indicates that birds, too, have a large-scale magnetic ‘map’.

### A ‘map’ based on components of the geomagnetic field

The idea of a magnetic ‘map’ initially met with less general skepticism than the magnetic compass, because magnetic intensity and inclination are forming gradients as is necessary for bi-coordinate navigation and also because no other suitable wide-spread environmental factors with gradients are known. A number of papers discussed a navigational ‘map’ from theoretical points of view, considering the available evidence for an involvement of magnetic factors in the navigation of various animals (e.g. Cain et al. 2005; Lohmann and Lohmann 2006; Freake et al. 2006 a.m.o.), some of them with critical notes (e.g. Wallraff 1999). Indeed, a ‘map’ based on magnetic total intensity and inclination only has its limitations. Boström and colleagues (2012) analyzed their worldwide distribution and showed that while there are considerable regions where the gradient directions of these two factors diverge more than 30°; there are others where they are more similar, at some places 2° or less, making navigation by the magnetic field alone difficult to impossible. In any case, there are numerous indications that the ‘map’, at least in birds, does not consist solely of magnetic factors, but is of multi-modal nature, including non-magnetic factors as well (see e.g. Walcott 2005; Beason and Wiltschko 2015).

There is also the problem of how accurately animals can measure intensity and inclination (see Komolkin et al. 2017). When pigeons were treated with a brief, strong magnetic pulse designed to interfere with the reception of magnetic ‘map’ information (see below), this affected their initial orientation only at several sites beyond 80 km from the loft (Beason et al. 1997). Apparently, magnetic cues are not used over short distances, probably because there must be a certain difference between the local values and the home values. But they seem to become important at greater distances, as the experiments of virtually displacing animals show.

The findings indicating an extended magnetic ‘map’ in animals as different as spiny lobsters, sharks, marine turtles and birds suggest that the use of a magnetic ‘map’ could be widespread among mobile animals. Future studies will maybe reveal further cases of magnetic navigation.

### The sensory basis

How animals record the magnetic ‘map’ factors, however, is still largely unknown. In birds, a second magnetic sense, based on magnetite, a special form of Fe_3_O_4_, is supported by their response to a brief, strong magnetic pulse (e.g. W. Wiltschko et al. 1994; Beason et al. 1997; Holland and Helm 2013). Magnetite-containing structures were described in the skin of the upper beak of birds (e.g. Fleissner et al 2003; Falkenberg et al. 2010), but were later claimed to be macrophages (Treiber et al 2012). However, as local anesthesia of the upper beak suppresses the pulse effect, they must be assumed to be located in that region (W. Wiltschko et al. 2009). The ‘map’ information is transmitted by the trigeminal nerve to the trigeminal brainstem complex (Heyers et al. 2010): Displacements—physically as well as magnetically simulated—could only be compensated if the reed warblers had intact trigeminal nerves (Kishkinev et al. 2013; Pakhomov et al. 2018).

How magnetic ‘map’ components are perceived in other animals is still unclear. Pulse-experiments with spiny lobsters and Chinook salmons, *Oncorhynchus tshawytscha*, are in agreement with receptors based on magnetite, but it is unclear to what extent their magnetic compass was likewise affected (Ernst and Lohmann 2016; Naisbett-Jones et al. 2020).

## Magnetic conditions as ‘sign posts’ or triggers

A further aspect of the magnetic field affecting animal behavior must be mentioned, although we do not know many examples yet, namely that specific magnetic conditions or changes in conditions may to act as ‘sign-posts’, triggering specific responses. These are spontaneous, obviously innate responses, in contrast to the navigational processes based on the learned ‘map’.

### Specific navigational responses

An indication for such a phenomenon could be the start of the second leg of migration in pied flycatchers, *Ficedula hypoleuca* (Muscicapidae*).* The central European populations of this species migrate southwest to Iberia, then change course to south or southeast to reach their winter quarters south of the Sahara. Birds kept in the geomagnetic field of Central Europe completed only the first part of the journey and then ceased to be active; birds for whom the magnetic field was gradually changed to the conditions in southern Spain, in contrast, continue to be active, now with southerly headings (Beck and Wiltschko 1988)—apparently, experiencing the change of the magnetic field was necessary to initiate the second part of migration.

A similar case was described for trans-equatorial migrating birds: Because of the avian magnetic compass is an inclination compass, the birds start out ‘equatorward’, but after crossing the magnetic equator, have to switch to ‘poleward’ to continue heading south. During autumn migration, garden warblers, *Sylvia borin*, showed southward tendencies, but after staying for three day in a horizontal magnetic field, headed northward, thus switching from ‘equatorward’ to ‘poleward’ (W. Wiltschko and Wiltschko 1992)—a response which in nature would have made them continuing southward. Obviously, being in a horizontal magnetic field triggers this change in magnetic heading.

Prominent cases of regional magnetic conditions acting as triggers that elicit specific responses are reported from marine animals. Hatchlings of loggerhead sea turtles in western Florida swim offshore into the Gulf Stream and spend their first years in the North Atlantic gyre. When hatchlings were tested in magnetic fields as they occur at several locations at the edge of the gyre, they showed spontaneous directional preferences that would have prevented them from leaving the gyre, e.g. from getting too far north or south (Lohmann et al 2001; Fuxjager et al. 2011 a.o). Here, spontaneous responses to certain magnetic condition ensure that the turtles stay in their favorable oceanic environment (see also Putman et al. 2014a). Similar responses were observed in two species of Pacific salmon, gen. *Oncorhynchus*—they, too, changed their headings when exposed to specific magnetic conditions, which, in nature, would have made them stay in their normal distribution area (Putman et al. 2014b, 2020).

### Triggering physiological responses

However, the trigger effect of specific magnetic conditions is not restricted to directional responses, but seems to include physiological aspects as well. For a group of thrush nightingales, *Luscinia luscinia* (Turdidae), the magnetic field conditions that they would encounter during migration until they reached Northern Egypt were simulated. Here the experimental birds increased their body mass considerably, much more than the control group that stayed in Swedish geomagnetic field (Fransson et al. 2001). The magnetic conditions in Northern Egypt act as sign post, triggering the refueling of migrants before they start crossing the ecological barrier of the Sahara desert. Experiments with European robins (Turdidae) and dunnocks, *Prunella modularis*, showed that the magnetic conditions affected the migration physiology differently, obviously adapted to the lengths and properties of the migration routes (e.g. Kullberg et al. 2007; Ilieva et al. 2018).

These examples show that the geomagnetic field is involved in variety of phenomena where it is advantageous that certain things happen at specific locations. Probably, these are not the only such cases; the ‘sign post’ function of magnetic conditions could be more wide-spread than is known today.

## Outlook

Research during the last 60 years has shown that a wide variety of animals use the geomagnetic field in various ways: as a compass and reference system, as components of a navigational ‘map’ and as ‘sign posts’ that not only control orientation, but also physiological functions, enabling birds to cover long distances. The geomagnetic field thus provides most helpful information for many aspects animal movements, and the animals make ample use of it. More examples will be discovered by future research.

## References

[CR1] Arendse MC, Barendregt A (1981). Magnetic orientation in the semi-terrestrial amphipod *Orchestia cavimana* and its interrelationship with photo-orientation and water loss. Physio Entomol.

[CR131] Arendse MC, Kruyswijk CJ (1981) Orientation of *Talitrus saltator* to magnetic fields. Neth J Sea Res 15:23–32

[CR2] Arendse MC, Vrins JCM (1975). Magnetic orientation and its relation to photic orientation in *Tenebrio molitor* L. (Coleoptera, Tenebrionidae). Netherl J Zool.

[CR3] August PV, Ayvazian SG, Anderson JGT (1989). Magnetic orientation in a small mammal, *Peromyscus leucopus*. J Mammal.

[CR4] Baker RR (1980). Goal orientation by blind-folded humans after long-distance displacement: possible involvement of a magnetic sense. Science.

[CR5] Baker RR (1987). Human navigation and magnetoreception: the Manchester experiments do replicate. Anim Behav.

[CR6] Baker RR, Mather JG (1982). Magnetic compass sense in the large yellow underwing moth, *Noctua Pronuba.* L. Anim Behav.

[CR7] Batschelet E (1965). Statistical methods for the analysis of problems in animal orientation and certain biological rhythms.

[CR8] Beason RC, Wiltschko W (2015). Cues indicating location in pigeon navigation. J Comp Physio A.

[CR9] Beason RC, Wiltschko R, Wiltschko W (1997). Pigeon homing: Effects of magnetic pulses on initial orientation. Auk.

[CR10] Beck W, Wiltschko W (1988) Magnetic factors control the migratory direction of Pied Flycatchers (*Ficedula hypoleuca* PALLAS). In: Ouellet H (Ed.), Acta XIX Congr. Intern. Ornithol., Ottawa 1986, Univ of Ottawa Press, Ottawa, pp 1955–1962

[CR11] Begall S, Malkemper EP, Cervaný J, Nemec P, Burda H (2013). Magnetic alignment in mammals and other animals. Mamm Biol.

[CR12] Berthold P (1988) The control of migration in European warblers. In: Ouellet H (Ed.), Acta XIX Congr. Intern. Ornithol, Ottawa 1986, Univ of Ottawa Press, Ottawa, pp251–249

[CR13] Boles LC, Lohmann KJ (2003). True navigation and magnetic maps in spiny lobsters. Nature.

[CR14] Bolte P, Einwich A, Seth PK, Chetverikova R, Heyers D, Wojahn I, Janssen-Bienhold U, Federle R, Hore P, Dedek K, Mouritsen H (2021) Cryptochrome 1a localisation in light- and dark-adapted retinae od several migratory and non-migratory birds species: no signs of light-dependent activation. Ethol Ecol Evol 33(3):248–272

[CR15] Bookman MA (1977). Sensitivity of the homing pigeon to an earth strength magnetic field. Nature.

[CR16] Boström JE, Åkesson S, Alerstam T (2012). Where on earth can animals use a geomagnetic bi-coordinate map for navigation?. Ecography.

[CR17] Burda H, Westenberger MSA, T, Wiltschko R, Wiltschko W, (1990). Evidence for magnetic compass orientation in the subterranean rodent *Cryptomys hottentotus* (Bathyergidae). Experientia.

[CR18] Cain SD, Boles LC, Wang JH, Lohmann KJ (2005). Magnetic orientation and navigation in marine turtles, lobsters and Molluscs: concepts and conundrums. Integr Comp Biol.

[CR19] Casamajor J (1927). Le Mystérieux ’sens de L’espace’. Rev Sc.

[CR20] Chernetsov N, Kishkinev D, Mouritsen H (2008). A long-distance avian migrant compensates for longitudinal displacement. Curr Biol.

[CR21] Davis L (1948). remarks on: “The physical basis of bird navigation”. J Appl Phys.

[CR22] DeJong D (1982). Orientation of comb building by honeybees. J Comp Physiol.

[CR23] Delius JD, Emmerton J (1978). Stimulus-dependent asymmetry in classical and instrumental discrimination learning by pigeons. Psychol Rec.

[CR24] Deutschlander ME, Philipps JB, Munro U (2012). Age-dependent orientation to magnetically simulated geographic displacement in migratory Australian silvereyes (*Zosterops l. lateralis*). Wilson J Ornithol.

[CR25] Emlen ST (1967). Migratory orientation in in the Indigo Bunting, *Passerina cyanea*. Auk.

[CR26] Emlen ST (1970). The influence of magnetic information on the orientation of the Indigo Bunting *Passerina Cyanea*. Anim Behav.

[CR27] Emlen ST, Wiltschko W, Demong NJ, Wiltschko R, Bergman S (1976). Magnetic direction findings: evidence for its use in migratory Indigo Bunting. Science.

[CR28] Ernst DA, Lohmann KJ (2016). Effect of magnetic pulses on Caribbean spiny lobsters: implications for magnetoreception. J Exp Biol.

[CR29] Falkenberg G, Fleissner G, Schuchard K, Kuehbacher M, Thalau P, Mouritsen H, Heyer D, Wellenreuter G, Fleissner G (2010). Avian magnetoreception: elaborate iron mineral containing dendrites in the upper beak seem to be a common feature of birds. PLoS ONE.

[CR30] Fischer JH, Freake MJ, Borland SC, Philipps JB (2001). Evidence for the use of magnetic map information by an amphibian. Anim Behav.

[CR31] Fleischmann PN, Grob B, Müller VL, Wehner R, Rössler W (2018). The geomagnetic field is a compass cue in *Cataglyphis* ant navigation. Curr Biol.

[CR32] Fleissner G, Holtkamp-Rötzler E, Hanzlik M, Winklhofer M, Fleissner G, Petersen N, Wiltschko W (2003). Ultrastructural analysis of a putative magnetoreceptor in the beak of homing pigeons. J Comp Neurol.

[CR33] Fransson T, Jakobsson S, Johansson P, Kullberg C, Lind J, Vallin A (2001). Magnetic cues trigger extensive refueling. Nature.

[CR34] Freake MJ, Muheim R, Phillips JB (2006). Magnetic maps in animals: a theory comes of age?. Quart Rev Biol.

[CR35] Freire R, Munro UH, Rogers LJ, Wiltschko R, Wiltschko W (2005). Chickens orient using a magnetic compass. Curr Biol.

[CR36] Fromme HG (1961). Untersuchungen über das Orientierungsvermögen nächtlich ziehender Kleinvögel (*Erithacus rubecula*, *Sylvia communis*). Z Tierpsychol.

[CR37] Fuxjager MJ, Eastwood BS, Lohmann KJ (2011). Orientation of hatchling loggerhead sea turtles to regional magnetic fields along a transoceanic migratory pathway. J Exp Biol.

[CR38] Gordon DA (1948). Sensitivity of the homing pigeon to the magnetic field of the earth. Science.

[CR39] Griffin DR (1952). Bird navigation. Biol Rev Cambridge Phil Soc.

[CR41] Griffin DR (1955) Bird navigation. In: Recent studies in Avian Biology. In: Wolfson A (Ed) Univ Il. Press, Urbana, pp 154–197)

[CR40] Griffin DR (1982). Ecology of migration: is magnetic orientation a reality?. Quart Rev Bio.

[CR42] Guerra PA, Gegear RJ, Reppert SM (2014). A magnetic compass aids monarch butterfly migration. Nature Comm.

[CR43] Henbest KB, Kukura P, Rodgers CT, Hore JP, Timmel CR (2004). Radio frequency magnetic field effects on a radical recombination reaction: A diagnostic test for the radical pair mechanism. J Am Chem Soc.

[CR44] Henshaw I, Fransson T, Jakobsson Kullberg C (2010). Geomagnetic field affects spring migratory direction in a long distance migrants. Behav Ecol Sociobiol.

[CR45] Heyers D, Zapka M, Hoffmeister M, Wild JM, Mouritsen H (2010). Magnetic field changes activate the trigeminal brainstem complex in a migratory bird. Proc Natl Acad Sci USA.

[CR46] Holland RA, Helm B (2013). A strong magnetic pulse affects the precvision of departure direction of naturally migrating adult but not juvenile birds. J R Soc Interface.

[CR47] Holland RA, Thorup K, Vonhof MJ, Cochan WW (2006). Bats orient using the Earth’s magnetic field. Nature.

[CR48] Holland RA, Kischvink JL, Doak TG, Wikelski M (2008). Bats use magnetite to detect the Earth’s magnetic field. PLoS ONE.

[CR49] Ilieva M, Biance G, Åkesson S (2018). Effect of geomagnetic field on migratory activity in a diurnal passerine migrant the dunnock *Prunella Modularis*. Anim Behav.

[CR50] Jacklyn PM (1992). “Magnetic” termite mounts’ surfaces are oriented to suit wind and shad conditions. Oecologia.

[CR51] Kalmijn AJ, Schmidt-Koenig K, Keeton WT (1978). Experimental evidence of geomagnetic orientation in elasmobranch fishes. Animal migration, navigation and homing.

[CR52] Karlsson J (1985). Behavioral responses of European silver eels (*Anguilla anguilla*) to the geomagnetic field. Helgol Meeresunters.

[CR53] Kavokin K, Chernetsov N, Pakhomov A, Bojarinova J, Kobylkov D, Namozov B (2014). Magnetic orientation of garden warblers (Sylvia borin) under 1.4 MHz radiofrequency magnetic fields. J R Soc Interface.

[CR54] Keller BA, Putman NF, Grubbs RD, Portney DS, Murphy TP (2021). Map-like use of Earth’s magnetic field in sharks. Curr Biol.

[CR55] Kishkinev D, Chernetsow N, Heyers D, Mouritsen H (2013). Migratory reed warblers need intact trigeminal nerves to correct for a 1000 km eastward displacement. PLoS One.

[CR56] Kishkinev D, Chernetsow N, Pakhomov A, Heyers D, Mouritsen H (2015). Eurasian reed warblers compensate for virtual magnetic displacement. Curr Biol.

[CR57] Komolkin AV, Kupriyanov P, Chudin A, Bojarinova J, Kavokin K, Chernetsov N (2017). Theoretically possible spatial accuracy of geomagnetic maps used by migrating animals. J R Soc Interface.

[CR58] Kramer G (1952). Experiments on bird orientation. Ibis.

[CR59] Kullberg C, Henshaw I, Jakobsson S, Johansson P, Fransson T (2007). Fuelling decisions in migratory birds: geomagnetic cues override the seasonal effect. Proc R Soc B.

[CR60] Light P, Salmon M, Lohmann KJ (1993). Geomagnetic orientation in Loggerhead Sea Turtles: evidence for a inclination compass. J Exp Biol.

[CR61] Lohmann KJ (1985). Geomagnetic field detection by the Western Atlantic spiny lobster, *Palinurus argus*. Mar Behav Physiol.

[CR62] Lohmann KJ (1991). Magnetic orientation by hatchling Loggerhead Sea Turtles (*Caretta caretta*). J Exp Biol.

[CR63] Lohmann KJ, Lohmann CMF (1993). A light-independent magentic compass in the Leatherback Sea Turtle. Biol Bull.

[CR64] Lohmann KJ, Lohmann CMF (2006). Sea turtles, lobsters and oceanic magnetic maps. Mar Freshw Behav Physiol.

[CR65] Lohmann KJ, Willows AOD (1987). Lunar-modulated geomagnetic orientation by a marine mollusk. Science.

[CR66] Lohmann KJ, Pentcheff ND, Nevitt GA, Stetten GD, Zimmer-Faust RK, Jarrard HE, Boles LC (1995). Magnetic orientation of spiny lobsters in the ocean: experiments with undersea coil systems. J Exp Biol.

[CR67] Lohmann KJ, Cain SD, Dodge SA, Lohmann CMF (2001). Regional magnetic fields as navigational markers for sea turtles. Science.

[CR68] Lohmann KJ, Lohmann CMF, Ehrhart LM, Bagley DA, Swing T (2004). Geomagnetic map use in sea-turtle navigation. Nature.

[CR69] Luschi P, Benhamou S, Girard C, Ciccioe S, Roos D, Sudre J, Benvenuti S (2007). Marine turtles use geomagnetic cues during open see homing. Curr Biol.

[CR70] Marhold S, Wiltschko W, Burda H (1997). A magnetic polarity compass for direction finding in a subterranean mammal. Naturwissenschaften.

[CR71] Marhold S, Burda H, Kreilos I, Wiltschko W (1997) Magnetic orientation in common mole-rats from Zambia. In: Orientation and navigation - birds, humans & other animals. Oxford. Royal Institute of Navigation, paper 5

[CR72] Mather JG, Baker RR (1981). Magnetic sense of direction in woodmice for route-based navigation. Nature.

[CR73] Mewaldt LR, Morton ML, Brown JL (1964). Orientation of migratory restlessness in *Zonotrichia*. Condor.

[CR74] Moore BR, Stanhope KJ, Wilox D (1987). Pigeons fail to detect low-frequency magnetic fields. Anim Learn Behav.

[CR75] Naisbett-Jones LC, Putman NF, Scanlan MM, Noakes DLG, Lohmann KJ (2020). Magnetoreception in fishes: the effect of magnetic pulses on orientation of juvenile Pacific salmon. J Exp Biol.

[CR76] Newton KC, Kajura SM (2020). The yellow stingray (*Urobatis jamaicensis*) can use magnetic field polarity to orient in space and solve a maze. Mar Biol.

[CR77] Nießner C, Denzau S, Gross J, Peichl L, Bischof HJ, Fleissner G, Wiltschko W, Wiltschko R (2011). Avian ultraviolet/violet cones identified as probable magnetoreceptors. PLoS ONE.

[CR78] Pakhomov A, Anashina A, Heyers D, Kobylkov D, Mouritsen H, Chernetsov N (2018). Magnetic map navigation in a migratory songbird requires trigeminal input. Scient Rep.

[CR79] Pardi L, Ercoloni A, Ferrara F, Scapini F (1985). Orientamento zonale solare e magnetico in Crostacei Anfipodi literali di regione equatoriali. Atti Accad- Lincei. Rend Sc Fis Mat Nat.

[CR80] Perdeck AC (1963). Does orientation without visual cues exist in robins?. Ardea.

[CR81] Phillips JB (1977). Use of the earth’s magnetic field by orienting cave salamanders (*Eurycea lucifuga*). J Comp Physiol.

[CR82] Phillips JB (1986). Magnetic compass orientation in the Eastern Red-spotted Newt (*Notophthalmus viridescens)*. J Comp Physiol A.

[CR83] Putman NF, Verley P, Endres CS, Lohmann KJ (2014). Magnetic navigation behavior and the oceanic ecology of young loggerhead sea turtles. J Exp Biol.

[CR84] Putman NF, Scanlan MN, Billman EJ, O’Neil JP, Couture RB, Quinn TP, Lohmann KJ, Noakes DL (2014). An inherited magnetic map guides ocean navigation in juvenile pacific salmon. Curr Biol.

[CR85] Putman N, Williams CR, Gallagher EP, Dittman AH (2020). A sense of place: pink salmon use a magnetic map for orientation. J Exp. Biol.

[CR130] Quinn TP (1980) Evidence for celestial and magnetic compass orientation in lake migrating Sockeye Salmon. J Comp Physiol 137:242–246

[CR86] Quinn TP, Merrill RT, Brannon EL (1981). Magnetic field detection in Sockeye Salmon. J Exp Bio.

[CR87] Reille A (1968). Essay de mise en èvidence d’une sensibilitè du pigeon au champ magnétique à l’aide d’une conditionnement nociceptive. J Pysiol (paris).

[CR88] Rickli M, Leuthold RH (1988). Homing in harvester termites: evidence of magnetic orientation. Ethology.

[CR92] Ritz T (2001) Disrupting magnetic compass orientation with radio frequency oscillating fields. In: Orientation & navigation—birds, humans & other animals. Proc 4th Intern Conf on Animal Navigation 2001. (Oxford, UK, St. Anne’s College), paper 4

[CR89] Ritz T, Adem S, Schulten K (2000). A model for photoreceptor-based magnetoreception in birds. Biophys J.

[CR90] Ritz T, Thalau P, Philllips JB, Wiltschko R, Wiltschko W (2004). Resonance effects indicate a radical-pair mechanism for avian magnetic compass. Nature.

[CR91] Ritz T, Wiltschko R, Hore PJ, Rodgers CT, Stapput K, Thalau P, Timmel CR, Wiltschko W (2009). Magnetic compass of birds is based on a molecule with optimal directional sensitivity. Biophys J.

[CR93] Sauer F (1957). Die Sternorientierung nächtlich ziehender Grasmücken (*Sylvia atricapilla, borin* und *curruca*). Z Tierpsychol.

[CR94] Schulten K, Windemuth A, Maret G, Boccara N, Kiepenheuer J (1986). Model for a physiological magnetic compass. Biophysical effects of steady magnetic fields.

[CR95] Shumakov ME (1990) The development of orientation capabilities of young nocturnal migrants under natural and experimental conditions. In: Viksne J, Vilks I (Eds) Baltic Birds (Riga) 5:146–149

[CR96] Souza JJ, Poluhowich JJ, Guerra RJ (1988). Orientation responses of American eels, *Anguilla rostrate,* to varying magnetic fields. Comp Biochem Physio.

[CR97] Stresemann E (1935). Haben die Vögel einen Ortssinn?. Ardea.

[CR98] Taylor PB (1987). Experimental evidence for geomagnetic orientation in juvenile salmon, *Onchorhynchus tschawytscha*. J Fish Biol.

[CR99] Treiber CD, Salzer MC, Riegler J, Edelmann N, Sugar C, Breuss M, Pichler P, Cadiou H, Saunders M, Shaw J, Keays DA (2012). Clusters of iron-rich cells in the upper beak of pigeons are macrophages not magneto-sensitive neurons. Nature.

[CR100] Vacha M, Byrne JH (2017). Magnetoreception in invertebrates. The Oxford handbook of invertebrate neurobiology.

[CR101] Vacha M, Drstová D, Půžova T (2008). *Tenebrio* beetles use magnetic inclination compass. Naturwissenschaften.

[CR102] Viguier C (1882). Le sens de l’orientation et ses organs chez les animaux et chez l’homme. Rev Phil France Etanger.

[CR103] von Middendorff A (1859). Die Isepiptesen Rußlands. Mém Acad Sc. S Petersbourg VI Ser Tome.

[CR104] Voss J, Keary N, Bischof HJ (2007). The use of the geomagnetic field for short-distance orientation in zebra finches. Behaviour.

[CR105] Walcott C, Schmidt-Koenig K, Keeton WT (1978). Anomalies in the Earth’s magnetic field increase the scatter in pigeons’ vanishing bearings. Animal migration, navigation and homing.

[CR106] Walcott C (2005). Multi-modal orientation cues in homing pigeons. Integr Comp Biol.

[CR107] Wallraff HG (1966). Versuche zur Frage der gerichteten Nachtzug-Aktivität von gekäfigten Singvögeln. Verh Dtsch Zool Ges Jena.

[CR108] Wallraff HG (1972). Nicht-visuelle Orientierung zugunruhiger Rotkehlchen (*Erithacus rubecula*). Z Tierpsychol.

[CR109] Wallraff HG (1974). Das Navigationssystem der Vögel.

[CR110] Wang Y, Pan Y, Parsons S, Walker MM, Zhang S (2007). Bats respond to polarity of a magnetic field. Proc R Soc B.

[CR1001] Wiltschko R, Wiltschko W (1978) Evidence for the use of magnetic outward journey information in homing pigeons. Naturwissenschaften 65, 112

[CR118] Wiltschko R, Wiltschko W (1996). Magnetoreception: why is conditioning so seldom successful?. Naturwissenschaften.

[CR119] Wiltschko R, Wiltschko W (2009). Avian Navigation. Auk.

[CR120] Wiltschko R, Wiltschko W (2014). Sensing magnetic directions in birds: radical pair processes involving cryptochrome. Biosensors.

[CR121] Wiltschko R, Wiltschko W (2015). Avian navigation: a combination of innate and learned mechanisms. Adv Study Behav.

[CR122] Wiltschko R, Wiltschko W (2019). Magnetoreception in birds. J R Soc Interface.

[CR111] Wiltschko W (1968). Über den Einfluß statischer Magnetfelder auf die Zugorientierung der Rotkehlchen (*Erithacus rubecula*). Z Tierpsychol.

[CR112] Wiltschko W, Gwinner E (1974). Evidence for an innate magnetic compass in Garden Warblers. Naturwissenschaften.

[CR113] Wiltschko W, Merkel FW (1966). Orientierung zugunruhiger Rotkehlchen im statischen Magnetfeld. Verh Dtsch Zool Ges Jena.

[CR114] Wiltschko W, Wiltschko R (1972). The magnetic compass of European Robins. Science.

[CR115] Wiltschko W, Wiltschko R (1975). The interaction of stars and magnetic field in the orientation system of night migrating birds. I. Autumn experiments with European Warblers (Gen. *Sylvia*). Z Tierpsychol.

[CR116] Wiltschko W, Wiltschko R (1975). The interaction of stars and magnetic field in the orientation system of night migrating birds. II. Spring experiments with European Robins (*Erithacus rubecula*). Z Tierpsychol.

[CR117] Wiltschko W, Wiltschko R (1992). Migratory orientation: magnetic compass orientation of Garden Warblers (*Sylvia bori*n) after a simulated crossing of the magnetic equator. Ethology.

[CR123] Wiltschko W, Wiltschko R, Keeton WT, Madden R (1983). Growing up in an altered magnetic field affects the initial orientation of young homing pigeons. Behav Ecol Sociobiol.

[CR124] Wiltschko W, Munro U, Beason RC, Ford H, Wiltschko R (1994). A magnetic pulse leads to a temporary deflection in the orientation of migratory birds. Experientia.

[CR125] Wiltschko W, Wiltschko R, Munro U, Ford H (1998). Magnetic versus celestial cues: cue-conflict experiments with migrating silvereyes at dusk. J Comp Physiol A.

[CR126] Wiltschko W, Munro U, Ford H, Wiltschko R (2009). Avian orientation: the pulse effect is mediated by the magnetite receptors in the upper beak. Proc R Soc B.

[CR127] Wilzeck C, Wiltschko W, Güntürkün O, Buschmann JO, Wiltschko R, Prior H (2010). Learning of magnetic compass directions in pigeons. Anim Cogn.

[CR128] Yeagley HL (1947). A preliminary study of a physical basis for bird navigation. J Appl Phys.

[CR129] Yeagley HL (1951). A preliminary study of a physical basis for bird navigation Part II. J Appl Phys.

